# Streptococcal Toxic Shock Syndrome Following Intestinal Obstruction in a Patient With Crohn’s Disease

**DOI:** 10.7759/cureus.100138

**Published:** 2025-12-26

**Authors:** Ayano Nishio, Masaya Iwamuro, Toshihiro Inokuchi, Mikako Ishiguro, Yuki Aoyama, Masahiro Takahara, Sakiko Hiraoka, Kohei Oguni, Hideharu Hagiya, Yuto Matsuoka, Tomoyuki Kanazawa, Motoyuki Otsuka

**Affiliations:** 1 Department of Gastroenterology and Hepatology, Okayama University Graduate School of Medicine, Dentistry, and Pharmaceutical Sciences, Okayama, JPN; 2 Department of Gastroenterology and Hepatology, Okayama University Hospital, Okayama, JPN; 3 Department of Infectious Diseases, Okayama University Hospital, Okayama, JPN; 4 Department of General Medicine, Okayama University Graduate School of Medicine, Dentistry, and Pharmaceutical Sciences, Okayama, JPN; 5 Department of Anesthesiology and Resuscitology, Okayama University Graduate School of Medicine, Dentistry, and Pharmaceutical Sciences, Okayama, JPN

**Keywords:** crohn’s disease (cd), group a streptococcus, immunosuppression, intestinal obstruction, streptococcal toxic shock syndrome (stss)

## Abstract

Streptococcal toxic shock syndrome (STSS) is a rare, life-threatening complication of invasive group A streptococcal (iGAS) infections. We report the case of a 24-year-old woman with Crohn's disease receiving immunosuppressive therapy who developed STSS following intestinal obstruction. On day 2, she developed fever, altered mental status, hypoxemia, erythema, and hypotension. Chest CT revealed bilateral pulmonary infiltrates, and blood cultures grew emm1-positive M1UK *Streptococcus pyogenes*, confirming STSS. Early multidisciplinary intervention resulted in rapid recovery without sequelae. This case emphasizes the importance of considering iGAS-induced STSS in septic shock, especially in immunocompromised patients, and highlights the need for prompt recognition and treatment.

## Introduction

Group A *Streptococcus* (GAS) is a gram-positive bacterium, *Streptococcus pyogenes*, that infects humans and causes a wide range of clinical conditions, including pharyngitis, scarlet fever, impetigo, cellulitis, and erysipelas. Beyond these superficial infections, GAS can occasionally invade sterile sites such as the bloodstream, deep soft tissues, or internal organs, leading to invasive GAS infections (iGAS) such as streptococcal toxic shock syndrome (STSS) and necrotizing fasciitis [1,2]. These invasive infections are associated with rapid disease progression, multiorgan failure, and high mortality, particularly when diagnosis and treatment are delayed [3].

STSS is one of the most severe manifestations of iGAS, occurring in approximately 13-15% of iGAS cases, with reported mortality rates ranging from 23% to 44% [4,5]. The syndrome is characterized by the sudden onset of shock and multiorgan failure due to massive cytokine release triggered by streptococcal superantigens [6]. Despite advances in critical care and antimicrobial therapy, the prognosis of STSS remains poor [7]. Therefore, prompt recognition of the clinical features and early initiation of aggressive therapy - typically combining high-dose penicillin and clindamycin - are essential to improve survival [8].

Recent epidemiological data have highlighted a global resurgence of iGAS and STSS cases since the COVID-19 pandemic, partly linked to the spread of highly virulent M1UK S. pyogenes lineages producing excessive superantigen toxins [9-12]. Immunocompromised patients, including those with autoimmune diseases or receiving biologic or immunosuppressive therapies, are particularly vulnerable to invasive infections due to impaired host defense mechanisms. In such individuals, GAS infections may present atypically and progress rapidly to septic shock or STSS [13].

In patients with inflammatory bowel disease, mucosal barrier dysfunction, intestinal ulceration, or obstruction may provide potential routes for bacterial invasion into the bloodstream. However, STSS secondary to intestinal pathology in Crohn’s disease has rarely been reported. Here, we describe the case of a young woman with Crohn’s disease receiving immunosuppressive therapy who developed STSS following intestinal obstruction. Early diagnosis and multidisciplinary management led to a favorable clinical outcome. This case highlights a unique route of bacterial invasion and underscores the importance of considering iGAS-induced STSS in immunocompromised patients presenting with sepsis or shock.

## Case presentation

**Figure 1:**
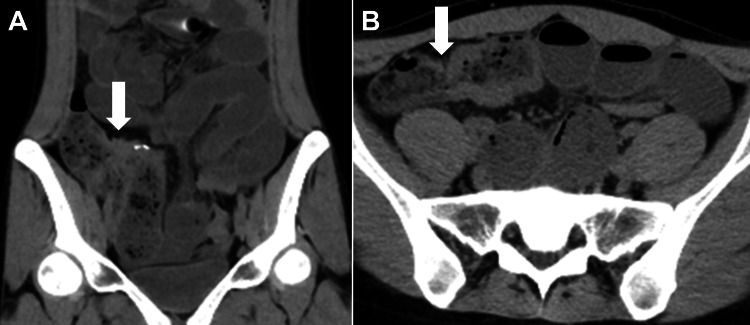
Abdominal computed tomography (A) Coronal computed tomography image showing proximal small bowel dilation and intraluminal fluid, with the postoperative anastomosis of the terminal ileum (white arrow) identified as the site of obstruction. (B) Axial computed tomography image showing small bowel wall thickening, with the postoperative anastomosis of the terminal ileum (white arrow) serving as the site of obstruction.

A 24-year-old Japanese woman presented with a chief complaint of abdominal pain. She had been receiving mesalazine 3,000 mg daily for Crohn's disease for the past eight years and adalimumab 40 mg weekly for the past seven years. Additionally, she had been taking colchicine 0.25 mg daily for nodular erythema for two years. Her abdominal pain gradually worsened after strawberry picking and was accompanied by vomiting, prompting her to visit the emergency department of our hospital in the evening. Abdominal computed tomography (CT) (Figure 1) revealed intestinal dilation originating from the anastomotic site following a previous ileocecal resection, leading to a diagnosis of intestinal obstruction and subsequent hospitalization.

Her medical history included Crohn’s disease (status post ileocecal resection at age 17) and acute epiglottitis. She reported occasional alcohol consumption but no history of smoking. She worked at an after-school daycare service, where she had daily contact with schoolchildren; however, no streptococcal outbreaks were reported at her workplace. The patient’s family had no significant medical history. On admission, she was alert (Japan Coma Scale I-1). She was 157.0 cm in height and weighed 43.6 kg. Her vital signs were as follows: temperature, 37.2°C; pulse, 85 beats per min, regular; blood pressure, 117/74 mmHg; respiratory rate, 18 breaths per min; and SpO₂, 97% on room air (Table 1). Heart and lung examinations were unremarkable. The abdomen was distended but soft, with tenderness in the upper abdomen and no rebound tenderness. No peripheral edema or cold extremities were observed.

**Table 1:** Vital signs

Vital signs	Patient records
Alertness	Japan Coma Scale I-1
Temperature	37.2°C
Blood pressure	117/74 mmHg
Pulse rate	85 beats per min
Respiratory rate	18 breaths per min
Pulse oximeter	97% on room air

Following admission, a nasogastric tube was inserted for decompression, and conservative management with fasting and intravenous fluids was initiated. However, her abdominal pain and vomiting persisted, and an upper ileal tube was placed later that day. Although her abdominal symptoms began to improve, she developed a fever that evening. Aspiration pneumonia secondary to ileal tube insertion was suspected, and empirical sulbactam/ampicillin therapy was initiated after cultures were obtained.

**Figure 2:**
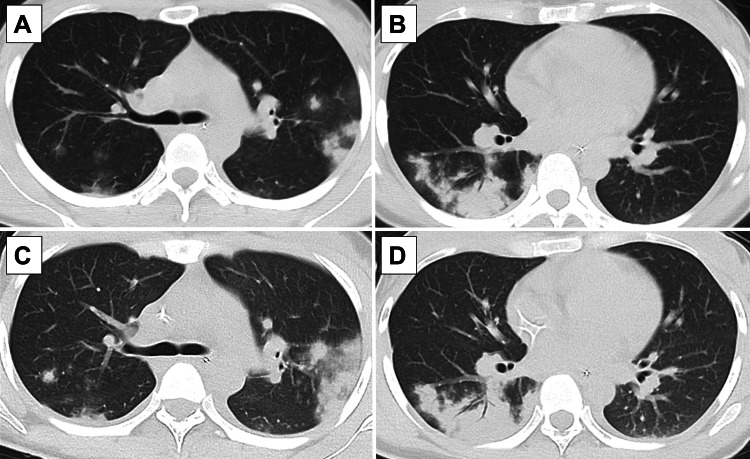
Chest computed tomography Computed tomography images reveal diffuse nodular opacities in both the lungs and infiltrative shadows with bronchial translucency in the right lower lobe. Some solid nodules display ring-shaped ground-glass opacities at their margins. Rapid progression of the infiltrative shadows was observed within a few hours (A, B: during oxygen desaturation on day 2 of hospitalization; C, D: after ICU admission).

Despite treatment, the patient developed a persistent high fever the following morning, accompanied by altered consciousness (JCS II-10) and hypoxemia (SpO₂ <90% on room air). Chest and abdominal CT (Figure 2) revealed diffuse nodular opacities in both lungs and infiltrative shadows with bronchial lucency in the right lower lobe, which had not been present the day before. Some nodules were surrounded by ground-glass opacities in a ring-like (halo) pattern. Laboratory tests (Table 2) performed on hospital day 2 revealed markedly elevated white blood cell counts and C-reactive protein (CRP). Results of the COVID-19 antigen test were negative. *S. pyogenes* was detected in both sets of blood cultures collected during the febrile period. Subsequently, the patient developed generalized erythema and hypotension, leading to a diagnosis of STSS and admission to the intensive care unit (ICU).

**Table 2:** Blood test results

Blood test results (units)	Patient value	Reference range
White blood cells (/μL)	15,990	3,300–8,600
Neutrophil (%)	91.4	40–70
Lymphocyte (%)	5.1	16.5–49.5
Eosinophils (%)	0.2	0.0–8.5
Basophils (%)	0.3	0.0–2.5
Monocytes (%)	3	2–10
Red blood cells (/μL)	4,400,000	4,350,000–5,550,000
Hemoglobin (g/dL)	12.6	13.7–16.8
Platelets (/μL)	214,000	158,000–348,000
Total protein (g/dL)	6.8	6.6–8.1
Albumin (g/dL)	3.3	4.1–5.1
Total bilirubin (mg/dL)	0.84	0.4–1.5
Blood urea nitrogen (mg/dL)	4.9	8.0–20.0
Creatinine (mg/dL)	0.67	0.65–1.07
Aspartate aminotransferase (U/L)	22	13–30
Alanine aminotransferase (U/L)	12	10–42
Alkaline phosphatase (U/L)	51	38–113
Lactate dehydrogenase (U/L)	223	124–222
Creatine kinase (U/L)	1881	59–248
Sodium (mmol/L)	137	138–145
Potassium (mmol/L)	3.2	3.6–4.8
Chloride (mEq/L)	103	101–108
Calcium (mg/dL)	8	8.8–10.1
C–reactive protein (mg/dL)	10.75	0–0.15
SARS-CoV-2 antigen	Negative	

**Figure 3:**
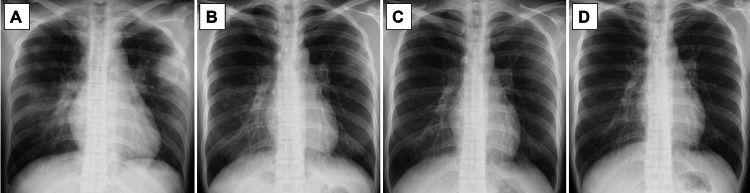
Chest radiography Chest radiography demonstrating progressive improvement of pulmonary infiltrates over time. (A) Chest radiography on day 1. (B) Chest radiography on day 5. (C) Chest radiography on day 10. (D) Chest radiography on day 12.

Fluid resuscitation and catecholamine support were administered in the ICU. Considering the patient's history of bacterial translocation associated with intestinal obstruction and her use of immunosuppressive medications, antimicrobial therapy with meropenem, penicillin G, clindamycin, and micafungin was added to the initial regimen. Her abdominal symptoms subsequently improved, and the ileal tube was removed. On hospital day 4 of hospitalization, norepinephrine was discontinued, and the patient was transferred to a general ward. She recovered uneventfully. Antibiotic therapy was de-escalated to ampicillin/sulbactam, and after repeated blood cultures confirmed bacterial clearance, she was switched to oral amoxicillin/clavulanate on hospital day 10. Chest radiography (Figure 3) showed further improvement, and she was discharged on hospital day 13 without any sequelae, ambulating independently. The total duration of antimicrobial therapy was 15 days.

Subsequently, the causative isolate was transferred to the National Institute of Infectious Diseases to identify the genetic determinants that contribute to virulence. Results indicated that the Sequence Type (ST) 1, M1UK lineage *S. pyogenes* harbored *emm1*, *speA*, *speB*, and *speF*.

## Discussion

Streptococcal toxic shock syndrome (STSS) is defined by the presence of shock accompanied by at least two manifestations such as hepatic or renal dysfunction, acute respiratory distress, disseminated intravascular coagulation, soft-tissue involvement, a diffuse erythematous rash, neurologic symptoms, or isolation of β-hemolytic streptococci from a normally sterile site (e.g., blood) [9]. In this case, GAS was detected in blood cultures, and the patient met the criteria for STSS, including systemic erythematous rash, central nervous system symptoms, laboratory abnormalities, and shock.

The global incidence of STSS has increased since the onset of the COVID-19 pandemic [10], and a similar trend has been reported in Japan [11,12]. Among the 6,666 cases reported in Japan between January 2015 and March 2024, 1402 were fatal, with more than half of the deaths occurring within three days of onset [14]. These findings underscore the importance of prompt diagnosis of STSS and initiation of early treatment with a combination of high-dose penicillin-class antibiotics and clindamycin.

GAS is primarily transmitted through direct contact with infected individuals. Children, immunocompromised persons, and elderly individuals are at the highest risk of GAS infections and their sequelae, with higher infection rates reported in schools, kindergartens, hospitals, and nursing facilities [13,15]. Although the patient described in this case report did not experience any GAS outbreaks at her workplace, she worked at an after-school day service. A systematic review from the United States reported that the prevalence of GAS carriage in healthy children without signs or symptoms of pharyngitis was 12% [16]. Therefore, it is possible that the patient’s risk of infection increased through contact with schoolchildren carrying GAS.

This case report illustrates STSS following intestinal obstruction in a patient with Crohn’s disease. Obstruction-induced ischemia and mucosal injury may facilitate bacterial translocation into the bloodstream, leading to bacteremia, sepsis, and potentially STSS. To our knowledge, a PubMed search using the terms “intestinal obstruction” or “ileus” in combination with “STSS” did not identify any similar cases. Although evidence is limited, a plausible mechanism involves intestinal obstruction causing ischemia and mucosal barrier disruption, followed by bacterial invasion, bacteremia, and the subsequent development of STSS.

The classification of GAS is based on the *emm* gene, which encodes the M protein, a well-established virulence factor. Among these, M1 strains of the emm1 type are the most commonly isolated. Since 2011, the prevalence of the M1UK lineage - defined by 27 characteristic single-nucleotide substitutions within M1 strains - has increased in the United Kingdom [12,17,18]. These strains reportedly produce approximately nine times more erythrogenic toxins and have higher transmissibility than non-UK lineage M1 strains [12,17]. In Japan, as of June 2024, 58.6% of the 377 GAS strains collected from patients with STSS were of the M1 type, of which 87.8% belonged to the M1UK lineage [9]. The rising prevalence of M1UK strains in Japan, along with their correlation with the increased incidence of GAS-related STSS, is a matter of concern. In the present patient, the isolate was characterized as belonging to the M1UK lineage and was positive for *speA*, *speB*, and *speF* but negative for *speC*. These genetic features, particularly the presence of multiple superantigen genes on an M1UK background, may have contributed to disease severity. Superantigens such as those encoded by *speA* and *speF* can trigger massive nonspecific T-cell activation and cytokine release, leading to toxic shock-like manifestations [6,19]. Moreover, the M1UK lineage has been shown to produce markedly higher levels of SpeA toxin and to spread more efficiently than conventional M1 strains. Taken together, the coexistence of multiple superantigen genes with an M1UK background provides a plausible explanation for the fulminant clinical course observed in this patient [18,20].

## Conclusions

We report the case of a young woman receiving immunosuppressive therapy for Crohn’s disease who developed STSS following intestinal obstruction. Prompt diagnosis based on clinical features and blood culture findings, together with early multidisciplinary treatment, resulted in a favorable outcome. This case underscores the importance of recognizing potentially life-threatening infections that can be prevented by early intervention and suspecting them based on the clinical course and findings.
